# Dexmedetomidine reduces in-hospital mortality in aneurysmal subarachnoid hemorrhage patients by modulating three key genes and inflammatory pathways: insights from clinical and bioinformatics analyses

**DOI:** 10.3389/fneur.2025.1554809

**Published:** 2025-07-24

**Authors:** Zhi-ang Li, Hong-cai Wang, Xue-wei Zhang, Li-hong Hu

**Affiliations:** ^1^Department of Anesthesiology, The Affiliated Lihuili Hospital of Ningbo University, Ningbo, China; ^2^Department of Neurosurgery, The Affiliated Lihuili Hospital of Ningbo University, Ningbo, China

**Keywords:** aneurysmal subarachnoid hemorrhage, dexmedetomidine, in-hospital mortality, inflammation, immune

## Abstract

**Background:**

Aneurysmal subarachnoid hemorrhage (aSAH) is a cerebrovascular disease with high mortality. Dexmedetomidine has a neuroprotective effect. This study aimed to explore the clinical and molecular association between dexmedetomidine and in-hospital mortality of aSAH.

**Methods:**

Patients with aSAH in the MIMIC-IV database were included and divided into non-in-hospital mortality and in-hospital mortality groups. Two machine learning algorithms random forest (RF) and XGBoost ranked treatment variables, and overlapping variables between these two algorithms were selected to evaluate their prognosis value for aSAH. Bioinformatics approaches, including DEG analysis, pathway enrichment, immune infiltration, and GSEA, explored potential mechanisms. Molecular docking assessed interactions between dexmedetomidine and identified hub genes.

**Results:**

A total of 505 individuals with aSAH were included in this study, with 114 dying in-hospital. Patients in the in-hospital mortality group exhibited older age, higher SAPS II scores, and altered physiological parameters. Dexmedetomidine was the most influential treatment variable, significantly associated with reduced in-hospital mortality. Bioinformatics identified three hub genes (MyD88, AR, AREG) related to aSAH and dexmedetomidine. These hub genes showed promising diagnostic accuracy in aSAH, with all AUC values over 0.67. Immune infiltration and GSEA highlighted the involvement of hub genes in inflammation and immune regulation. Molecular docking revealed AR as a direct target of dexmedetomidine (binding energy = −5.68 kcal/mol).

**Conclusion:**

Dexmedetomidine is correlated with reduced in-hospital mortality in aSAH, potentially by regulating AR and immune pathways. These findings highlight AR as a promising therapeutic target of dexmedetomidine for aSAH management.

## 1 Introduction

Aneurysmal subarachnoid hemorrhage (aSAH) is a severe cerebrovascular event caused by subarachnoid hemorrhage (SAH) due to the rupture of intracranial aneurysms (1). aSAH accounts for 2–7% of all strokes, with an incidence of 9.1 cases per 100,000 individuals (2). The incidence of aSAH peaks between the ages of 50–60 years, and females have a 1.6 times higher risk than males (3). Despite advances in treatment, such as endovascular coil embolization, microsurgery, and management of postoperative complications, 12% of aSAH patients die before reaching the hospital, and the in-hospital mortality rate for treated aSAH patients ranges from 8.3 to 66.7% (4, 5). Survivors often experience long-term neurological impairments, significantly impacting their quality of life (6). Currently, the treatment of aSAH primarily focuses on early aneurysm repair, prevention of rebleeding, control of intracranial pressure, and management of delayed cerebral ischemia and vasospasm (4). However, secondary pathological processes such as neuroinflammation, disruption of the blood–brain barrier, and oxidative stress continue to severely impact patient outcomes (3). This underscores the urgent need for accurate prediction of clinical prognosis to guide potential progression and therapeutic strategies for aSAH. Due to its multiple pharmacological properties, dexmedetomidine holds promise as a potential therapeutic intervention to mitigate these secondary injuries.

Dexmedetomidine is a highly selective α2-adrenergic receptor agonist. By blocking α-receptors in the brainstem, it inhibits central sympathetic outflow, suppresses norepinephrine release, and exerts sedative and analgesic effects (7). Consequently, it is widely used in intensive care and anesthetic management (8). Unlike traditional sedatives, dexmedetomidine minimizes respiratory depression while providing sedation (9). Additionally, it possesses anti-inflammatory, antioxidant, and immunomodulatory properties, demonstrating neuroprotective effects in various neurological conditions (10). Studies suggest that dexmedetomidine may be a protective therapy for SAH patients by maintaining intracranial homeostasis, repairing the damaged blood–brain barrier, and preventing vasospasm (11). In animal experiments, intraperitoneal injection of dexmedetomidine at a dose of 25 μg/kg significantly reduced neutrophil infiltration, microglial activation, and the release of pro-inflammatory cytokines, while improving neurological scores and the expression of tight junction proteins (12). Kallioinen et al. found that higher doses of dexmedetomidine (0.7–1 and 1.4 μg/kg/h) were associated with impaired dynamic cerebral autoregulation, as indicated by a decreased transient hyperemic response ratio (13). In addition, a randomized controlled trial showed that a high-dose dexmedetomidine regimen—an initial intravenous infusion of 0.5 μg/kg over 10 min followed by a continuous intraoperative infusion of 0.4 μg/kg/h—significantly reduced total nimodipine consumption within 48 h postoperatively and promoted early recovery in aSAH patients (14). Despite these findings, the specific mechanisms of dexmedetomidine in aSAH patients and its impact on clinical prognosis remain insufficiently elucidated.

By integrating clinical data, machine learning algorithms, and bioinformatics analysis, we explored key factors associated with in-hospital mortality of aSAH patients and delved into the molecular mechanisms underlying this association. This study aimed to investigate the clinical and molecular relationship between dexmedetomidine use and the prognosis of aSAH patients, laying the foundation for optimizing treatment strategies for aSAH.

## 2 Methods

### 2.1 Study population

This study retrieved data from the Medical Information Mart for Intensive Care IV (MIMIC-IV), a large-scale database developed and maintained by the Laboratory for Computational Physiology at the Massachusetts Institute of Technology. The database contains detailed medical information from patients admitted to the intensive care unit (ICU) of Beth Israel Deaconess Medical Center (15). As the database does not include protected health information and all patient data are anonymized, informed consent was waived. Patients diagnosed with SAH were identified based on the International Classification of Diseases, Ninth and Tenth Revision (ICD-9 and ICD-10) codes, resulting in 2,808 patients. The following exclusion criteria were applied: (1) traumatic SAH (*n* = 53); (2) lack of surgical intervention (*n* = 1,057); (3) incomplete sedative drug records (*n* = 1,150); (4) length of ICU stays<1 day (*n* = 43).

### 2.2 Clinical data collection and outcomes

Relevant clinical information was extracted using structured query language (SQL). The collected variables were categorized into six groups: (1) Demographics, including age, gender, body mass index (BMI), and marriage status; (2) Comorbidities, such as hyperlipidemia, diabetes, and coronary heart disease; (3) Clinical severity, including Glasgow coma scale (GCS), World Federation of Neurosurgical Societies (WFNS) grade, and Simplified Acute Physiology Score II (SAPS II); (4) Vital signs, including heart rate, systolic blood pressure (SBP), diastolic blood pressure (DBP), and percutaneous oxygen saturation (SpO_2_); (5) Laboratory tests, such as white blood cell (WBC) count, platelet count, creatinine, international normalized ratio (INR), prothrombin time (PT), partial thromboplastin time (PTT), neutrophils, monocytes, lymphocytes, sodium, potassium, and bicarbonate; (6) Treatments, including surgical type and administration of propofol, midazolam, or dexmedetomidine. The primary outcome of the study was in-hospital mortality.

### 2.3 Bioinformatics data collection and preprocessing

Two SAH-related microarray datasets (GSE13353 and GSE54083) were obtained from the Gene Expression Omnibus (GEO) database (https://www.ncbi.nlm.nih.gov/geo/). After removing batch effects using the “COMBAT” algorithm, the merged expression profiles comprised 19 aSAH samples and 13 control samples. Dexmedetomidine-related target genes were retrieved from the GeneCards database (https://www.genecards.org/).

### 2.4 Differential expression analysis

Differentially expressed genes (DEGs) between the aSAH and control groups were identified using the R package “limma,” with thresholds set at |fold change| ≥ 1.5 and *p* < 0.05. A Venn diagram was used to identify the intersection of DEGs and dexmedetomidine-related target genes for further analysis.

### 2.5 Functional enrichment analysis

Potential functions of DEGs and dexmedetomidine-related target genes were explored through Kyoto Encyclopedia of Genes and Genomes (KEGG) pathway analysis. Gene set enrichment analysis (GSEA) was conducted to identify pathways associated with hub genes. KEGG analysis was performed using the “clusterProfiler” package, while GSEA was executed with the “org. Hs.eg.db” and “GSEABase” packages.

### 2.6 Immune infiltration analysis

The “CIBERSORT” method was employed to estimate the fraction of 22 immune cell types in the aSAH and control groups. Boxplots and correlation heatmaps were generated to visualize the results.

### 2.7 Statistical analysis

For clinical characteristics, continuous variables with a normal distribution were expressed as mean±standard deviation and compared between groups using the Kolmogorov–Smirnov test. Non-normally distributed variables were presented as median [interquartile range (IQR)] and analyzed using the Mann–Whitney U test or Kruskal-Wallis test. Categorical variables were reported as percentages [n (%)] and compared using the chi-square test. Random forest (RF) and XGBoost algorithms were employed to rank variables with significant intergroup differences by importance. The intersection of the top five ranked variables from both algorithms was used to construct receiver operating characteristic (ROC) curves, which evaluated the predictive performance of the selected variables for in-hospital mortality in aSAH patients. Statistical differences in area under curve (AUC) values of these variables were assessed using the DeLong test. Additionally, collinearity analysis was conducted for variables with significant intergroup differences, retaining those with a variance inflation factor (VIF) < 3 to eliminate potential confounders. Logistic regression analysis was performed to investigate the association between dexmedetomidine and in-hospital mortality in aSAH patients, with adjustments applied as follows: Model 1 adjusted for creatinine, PTT, neutrophils, lymphocytes, and platelets; Model 2 adjusted for coronary heart disease and diabetes; and Model 3 adjusted for age, SpO_2_, SAPS II, and DBP.

In bioinformatics analyses, the clinical diagnostic value of hub genes for aSAH was demonstrated using ROC and decision curve analysis (DCA). Pearson correlation analysis was conducted to evaluate the relationships between hub genes and 22 immune cell types.

Statistical analyses were conducted using SPSS 22.0 and R Studio 4.2.3, with a two-sided *p* < 0.05 considered statistically significant.

## 3 Results

### 3.1 Baseline characteristics of the study population

Table 1 presents the baseline characteristics of the study population. A total of 505 aSAH patients were included in the study, categorized into the non-in-hospital mortality group (*n* = 391) and the in-hospital mortality group (*n* = 114). The median age of patients in the non-in-hospital mortality group was 58 years, while that in the in-hospital mortality group was 68 years, showing a significant intergroup difference (*p* < 0.001). The non-in-hospital mortality group included 168 females (42.967%), and the in-hospital mortality group included 52 females (45.614%), with no statistically significant difference between the groups. Similarly, no significant differences were observed between the groups in terms of BMI and marital status (*p* > 0.05). In the non-in-hospital mortality group, 74 patients (18.926%) had diabetes, and 28 (7.161%) had coronary heart disease, whereas in the in-hospital mortality group, the respective data were 35 (30.702%) and 18 (15.789%). These differences were statistically significant (*p* < 0.01). Additionally, the in-hospital mortality group had significantly higher SAPS II scores (*p* < 0.001). Compared to the non-in-hospital mortality group, patients in the in-hospital mortality group had higher levels of SpO₂, creatinine, INR, PT, PTT, and neutrophils, while exhibiting lower levels of DBP, platelets, and lymphocytes (*p* < 0.05).

**Table 1:** Baseline characteristics of the study population.

Variable categories	Variables		Total (*n* = 505)	Non-in-hospital mortality (*n* = 391)	In-hospital mortality (*n* = 114)	*p*
Demographics	Age (year), median [IQR]		60.000 [48.000, 72.000]	58.000 [47.000, 69.000]	68.000 [57.000, 78.000]	<0.001
	BMI (kg/m^2^), median [IQR]		24.200 [21.300, 28.300]	24.400 [21.300, 28.300]	23.500 [19.600, 26.300]	0.206
	Gender, *n* (%)	Female	220 (43.564)	168 (42.967)	52 (45.614)	0.616
		Male	285 (56.436)	223 (57.033)	62 (54.386)	
	Marriage status, *n* (%)	Married	187 (49.734)	150 (49.342)	37 (51.389)	0.755
		Single	189 (50.266)	154 (50.658)	35 (48.611)	
Comorbidities	Hyperlipidemia, *n* (%)	No	368 (72.871)	287 (73.402)	81 (71.053)	0.620
		Yes	137 (27.129)	104 (26.598)	33 (28.947)	
	Diabetes, *n* (%)	No	396 (78.416)	317 (81.074)	79 (69.298)	0.007
		Yes	109 (21.584)	74 (18.926)	35 (30.702)	
	Coronary heart disease, *n* (%)	No	459 (90.891)	363 (92.839)	96 (84.211)	0.005
		Yes	46 (9.109)	28 (7.161)	18 (15.789)	
Clinical severity	GCS, median [IQR]		9.000 [6.000, 13.000]	9.000 [7.000, 13.000]	8.000 [3.000, 15.000]	0.227
	WFNS grade, *n* (%)	I–III	155 (30.693)	116 (29.668)	39 (34.211)	0.355
		IV–V	350 (69.307)	275 (70.332)	75 (65.789)	
	SAPS II, median [IQR]		34.000 [27.000, 42.000]	33.000 [26.000, 40.000]	39.000 [34.000, 49.000]	<0.001
Vital signs	Heart rate (bears/min), median [IQR]		66.000 [58.000, 77.000]	66.000 [58.000, 77.000]	66.000 [58.000, 76.000]	0.700
	SBP (mmHg), mean ± SD		127.029 ± 21.913	127.946 ± 21.775	123.650 ± 22.090	0.121
	DBP (mmHg), median [IQR]		63.000 [54.000, 72.000]	63.000 [56.000, 73.000]	60.000 [51.000, 67.000]	0.009
	SpO_2_, median [IQR]		85.000 [77.000, 85.000]	85.000 [82.000, 85.000]	74.000 [50.000, 85.000]	<0.001
Laboratory tests	WBC (K/μL), median [IQR]		11.400 [8.900, 14.400]	11.400 [8.800, 14.100]	11.900 [9.800, 16.200]	0.100
	Platelet (K/μL), median [IQR]		186.000 [140.000, 241.000]	190.000 [144.000, 242.000]	169.000 [125.000, 230.000]	0.015
	Creatinine(mg/dL), median [IQR]		0.800 [0.600, 1.100]	0.800 [0.600, 1.000]	1.000 [0.700, 1.500]	<0.001
	INR, median [IQR]		1.100 [1.000, 1.200]	1.100 [1.000, 1.200]	1.200 [1.100, 1.300]	<0.001
	PT (s), median [IQR]		12.100 [11.200, 13.200]	11.900 [11.200, 12.900]	12.600 [11.600, 14.000]	<0.001
	PTT (s), median [IQR]		25.900 [23.900, 28.300]	25.800 [23.700, 28.100]	26.700 [24.400, 29.500]	0.012
	Neutrophil (K/μL), median [IQR]		74.100 [65.600, 81.600]	73.400 [65.100, 80.700]	79.400 [68.000, 85.000]	0.011
	Monocyte (K/μL), median [IQR]		4.100 [1.000, 6.900]	4.200 [1.000, 6.900]	4.000 [2.000, 6.000]	0.909
	Lymphocyte (K/μL), median [IQR]		7.400 [5.000, 12.100]	8.000 [5.500, 13.000]	5.400 [3.000, 8.600]	<0.001
	Sodium (mmol/L), median [IQR]		132.000 [81.000, 136.000]	132.000 [86.000, 136.000]	130.000 [62.000, 137.000]	0.862
	Potassium (mmol/L), median [IQR]		3.200 [3.000, 3.500]	3.200 [3.000, 3.400]	3.300 [2.900, 3.700]	0.358
	Bicarbonate (mmol/L), median [IQR]		19.000 [17.000, 21.000]	19.000 [17.000, 21.000]	18.000 [16.000, 21.000]	0.058

BMI, body mass index; SBP, systolic blood pressure; DBP, diastolic blood pressure; SpO_2_, percutaneous oxygen saturation; WBC, white blood cell; INR, international normalized ratio; PT, prothrombin time; PTT, partial thromboplastin time; GCS, Glasgow coma scale; WFNS, World Federation of Neurosurgical; SAPS II, simplified acute physiology score II; IQR, interquartile range; SD, standard deviation.

### 3.2 Dexmedetomidine is an important variable for patients with aSAH

We also investigated the treatments received by the study population, as shown in Table 2. A total of 68 patients underwent endovascular intervention for aneurysm, with 55 (14.066%) in the non-in-hospital mortality group and 13 (11.404%) in the in-hospital mortality group. Significant differences were observed in the usage of anesthetic agents between the two groups. Specifically, the number of patients receiving dexmedetomidine, propofol combined with dexmedetomidine, and midazolam combined with dexmedetomidine differed significantly (*p* < 0.05).

**Table 2:** Treatments received by the study population.

Treatments		Total (*n* = 505)	Non-in-hospital mortality (*n* = 391)	In-hospital mortality (*n* = 114)	*p*
Surgery type, *n* (%)	Endovascular intervention for aneurysm	68 (13.465)	55 (14.066)	13 (11.404)	0.464
	Others	437 (86.535)	336 (85.934)	101 (88.596)	0.146
Propofol, *n* (%)	No	216 (42.772)	174 (44.501)	42 (36.842)	0.146
	Yes	289 (57.228)	217 (55.499)	72 (63.158)	
Midazolam, *n* (%)	No	270 (53.465)	215 (54.987)	55 (48.246)	0.204
	Yes	235 (46.535)	176 (45.013)	59 (51.754)	
Dexmedetomidine, *n* (%)	No	266 (52.673)	185 (47.315)	81 (71.053)	<0.001
	Yes	239 (47.327)	206 (52.685)	33 (28.947)	
Propofol+Midazolam, *n* (%)	No	478 (94.653)	375 (95.908)	103 (90.351)	0.020
	Yes	27 (5.347)	16 (4.092)	11 (9.649)	
Propofol+Dexmedetomidine, *n* (%)	No	401 (79.406)	303 (77.494)	98 (85.965)	0.049
	Yes	104 (20.594)	88 (22.506)	16 (14.035)	
Midazolam+Dexmedetomidine, *n* (%)	No	466 (92.277)	355 (90.793)	111 (97.368)	0.021
	Yes	39 (7.723)	36 (9.207)	3 (2.632)	
Propofol+Midazolam+Dexmedetomidine, *n* (%)	No	461 (91.287)	357 (91.304)	104 (91.228)	0.980
	Yes	44 (8.713)	34 (8.696)	10 (8.772)	
Ventilation status, *n* (%)	No	23 (4.554)	17 (4.348)	6 (5.263)	0.680
	Yes	482 (95.446)	374 (95.652)	108 (94.737)	

**Figure 1:**
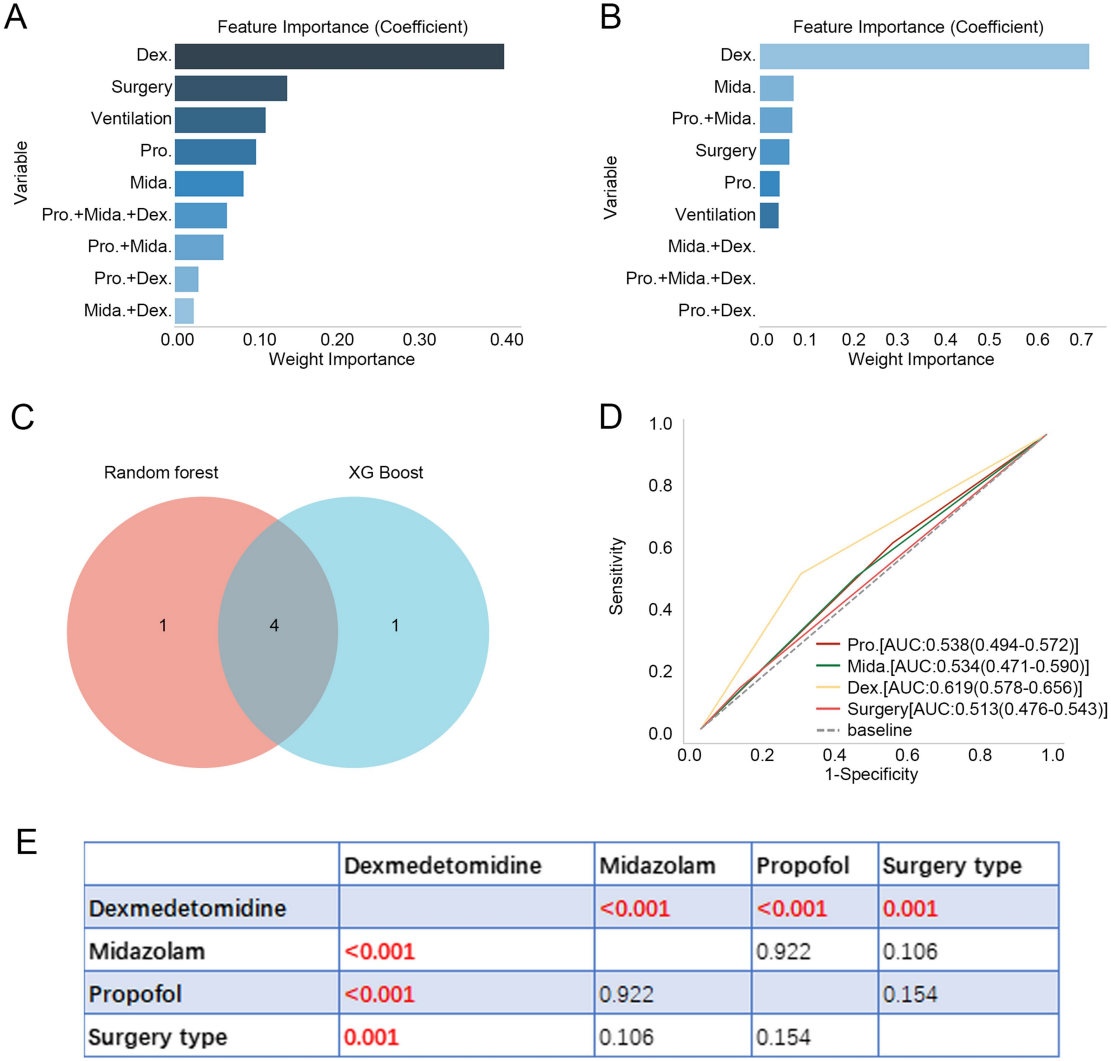
Dexmedetomidine is an important variable for patients with aSAH. **(A,B)** Feature importance of treatment-related variables in RF and XG Boost algorithms. **(C)** Four intersecting variables were identified between RF and XG Boost algorithms. **(D)** ROC curve revealed the predictive value of four intersecting variables for in-hospital mortality in patients with aSAH. **(E)** Difference in AUC values of four intersecting variables. Dex.: dexmedetomidine, Pro.: propofol, Mida.: midazolam. aSAH, aneurysmal subarachnoid hemorrhage; RF, random forest; ROC, receiver operator characteristic; AUC, area under curve.

Additionally, we ranked the importance of treatment-related variables using the RF and XG Boost algorithms. As shown in Figures 1A,B, dexmedetomidine ranked as the most important variable in both algorithms. By taking the intersection of the top five variables identified by each algorithm using a Venn diagram, four common variables were identified: dexmedetomidine, midazolam, propofol, and surgery type (Figure 1C). Subsequently, we explored the predictive value of these variables for in-hospital mortality in aSAH using ROC curves. Dexmedetomidine exhibited the highest AUC value at 0.619 (Figure 1D). Furthermore, differences in AUC values among the four variables revealed that the AUC value for dexmedetomidine was significantly higher than those of the other variables (*p* < 0.01, Figure 1E).

### 3.3 Dexmedetomidine is associated with in-hospital mortality of patients with aSAH

Based on the above findings, we further investigated the association between dexmedetomidine use and in-hospital mortality among aSAH patients. Variables identified as significant in univariate analysis were subjected to collinearity analysis, and those with a VIF > 3 were excluded (Table 3). The remaining variables were included in subsequent logistic regression analyses. After adjusting for various confounding factors, dexmedetomidine use was consistently and significantly associated with a reduction in in-hospital mortality among aSAH patients (Table 4). The odds ratios (OR) for dexmedetomidine were as follows: Model 1: OR = 0.497 (95% CI: 0.262–0.923, *p* = 0.029), Model 2: OR = 0.357 (95% CI: 0.223–0.559, *p* < 0.001), and Model 3: OR = 0.460 (95% CI: 0.252–0.820, *p* = 0.009).

**Table 3:** Collinearity analysis.

Variables	Variance inflation factor
INR	19.606
PT	19.295
SAPS II	1.573
Age	1.394
Neutrophil	1.279
PTT	1.277
Creatinine	1.26
Lymphocyte	1.226
DBP	1.172
Coronary heart disease	1.158
Platelet	1.126
SpO_2_	1.123
Diabetes	1.122

INR, international normalized ratio; PT, prothrombin time; SAPS II, simplified acute physiology score II; PTT, partial thromboplastin time; DBP, diastolic blood pressure; SpO_2_, percutaneous oxygen saturation.

**Table 4:** Dexmedetomidine is associated with in-hospital mortality of patients with aSAH.

Variable	OR (95%CI), *p*		
	Model 1	Model 2	Model 3
Dexmedetomidine	0.497 (0.262–0.923), 0.029	0.357 (0.223–0.559), <0.001	0.460 (0.252–0.820), 0.009

Model 1: adjusted for creatinine, PTT, neutrophil, lymphocyte, and platelet; Model 2: adjusted for coronary heart disease and diabetes; Model 3: adjusted for age, SpO_2,_ SAPS II, and DBP.

aSAH, aneurysmal subarachnoid hemorrhage; OR, odds ratio; CI, confidence interval.

### 3.4 Three hub genes related to aSAH and dexmedetomidine were identified

**Figure 2:**
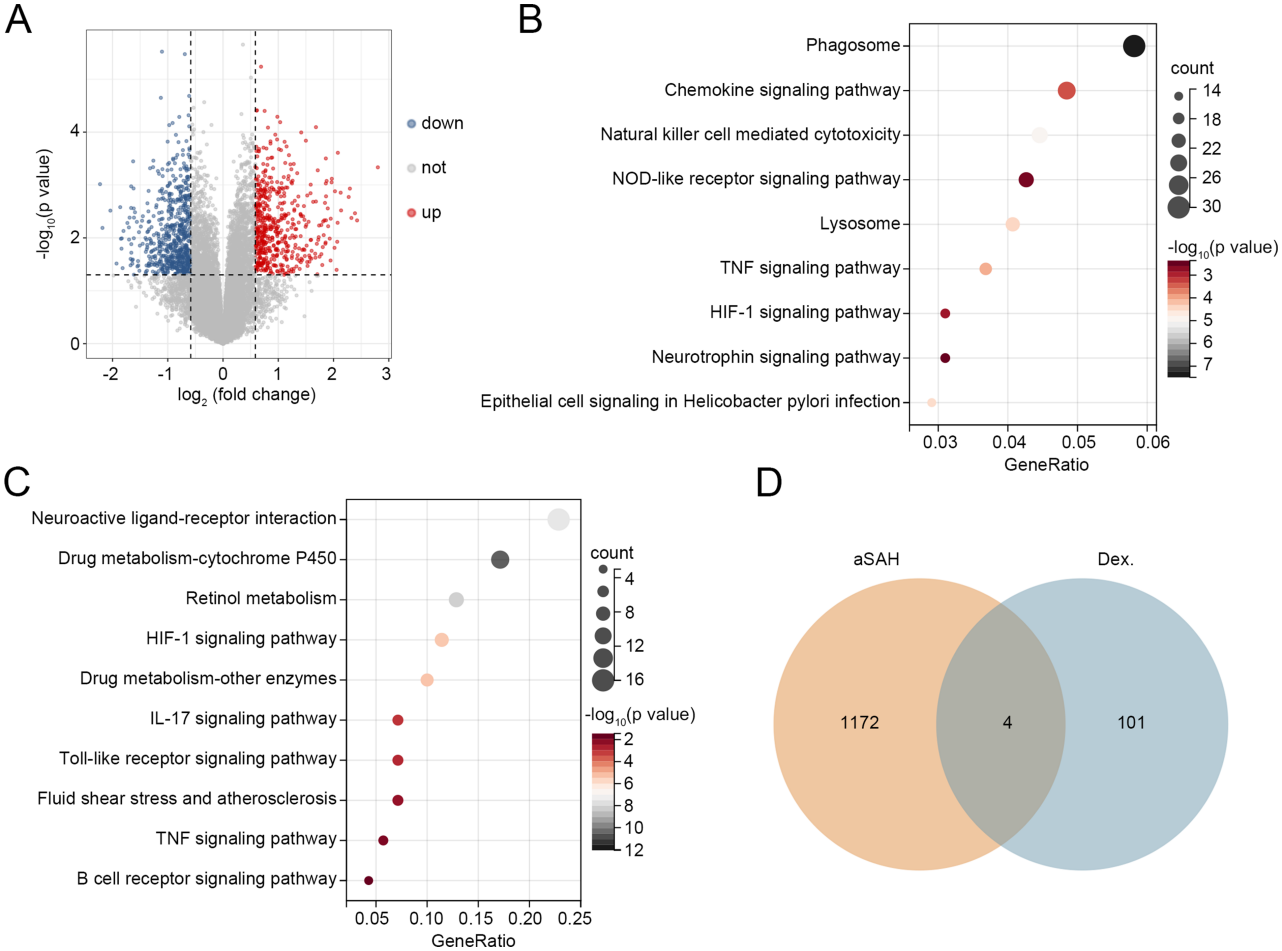
Three hub genes related to aSAH and dexmedetomidine were identified. **(A)** Volcano plot of DEGs related to aSAH. **(B)** KEGG pathways related to DEGs. **(C)** KEGG pathways related to targets of dexmedetomidine. **(D)** Overlapping genes between aSAH-related DEGs and targets of dexmedetomidine. Dex.: dexmedetomidine. aSAH, aneurysmal subarachnoid hemorrhage; DEGs, differentially expressed genes; KEGG, Kyoto Encyclopedia of Genes and Genomes‌.

To further investigate the potential mechanisms by which dexmedetomidine reduces in-hospital mortality among aSAH patients, bioinformatics analyses were performed. In aSAH-related GEO datasets, we identified 529 upregulated DEGs and 647 downregulated DEGs (Figure 2A). These DEGs were enriched in pathways such as the chemokine signaling pathway, NOD-like receptor signaling pathway, TNF signaling pathway, HIF-1 signaling pathway, and neurotrophin signaling pathway (Figure 2B). Additionally, in the GeneCards database, we identified 105 dexmedetomidine-related targets. KEGG pathway analysis revealed that these targets were enriched in the HIF-1 signaling pathway, IL-17 signaling pathway, Toll-like receptor signaling pathway, TNF signaling pathway, and B cell receptor signaling pathway (Figure 2C). By intersecting the 1,176 DEGs with the 105 dexmedetomidine-related targets, we identified four overlapping genes: myeloid differentiation primary response protein 88 (MyD88), androgen receptor (AR), FGD5-AS1, and amphiregulin (AREG) (Figure 2D). Since FGD5-AS1 is a lncRNA, it was excluded from subsequent analyses. The remaining three genes were considered hub genes associated with both aSAH and dexmedetomidine.

**Figure 3:**
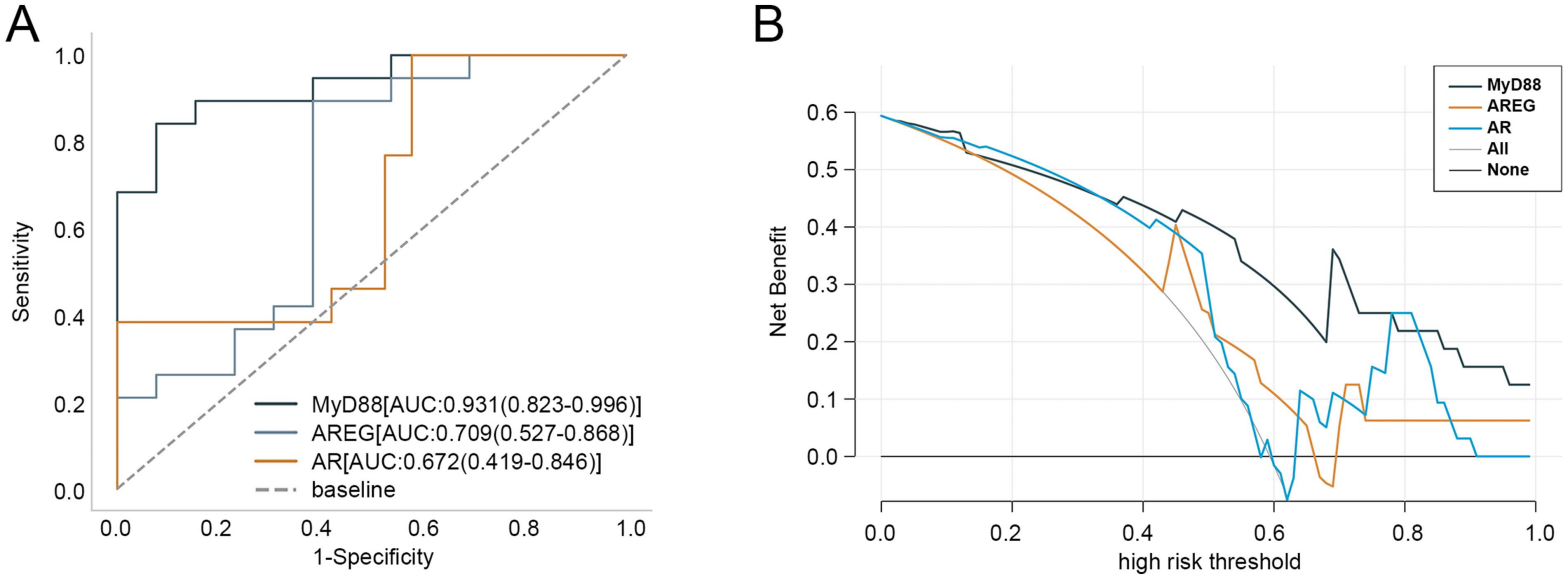
Clinical value of three hub genes in aSAH. **(A)** ROC curves of three hub genes in diagnosing aSAH. **(B)** DCA curve of three hub genes. aSAH, aneurysmal subarachnoid hemorrhage; ROC, receiver operator characteristic; DCA, decision curve analysis.

Next, we evaluated the diagnostic performance of these hub genes for aSAH using ROC curves. As shown in Figure 3A, MyD88 demonstrated the highest diagnostic accuracy with an AUC value of 0.931, followed by AREG and AR, with AUC values of 0.709 and 0.672, respectively. DCA curve revealed that MYD88 consistently provided the highest net benefit across most high-risk thresholds, with AR ranking second (Figure 3B).

### 3.5 Association of hub genes with immune cells in aSAH

**Figure 4:**
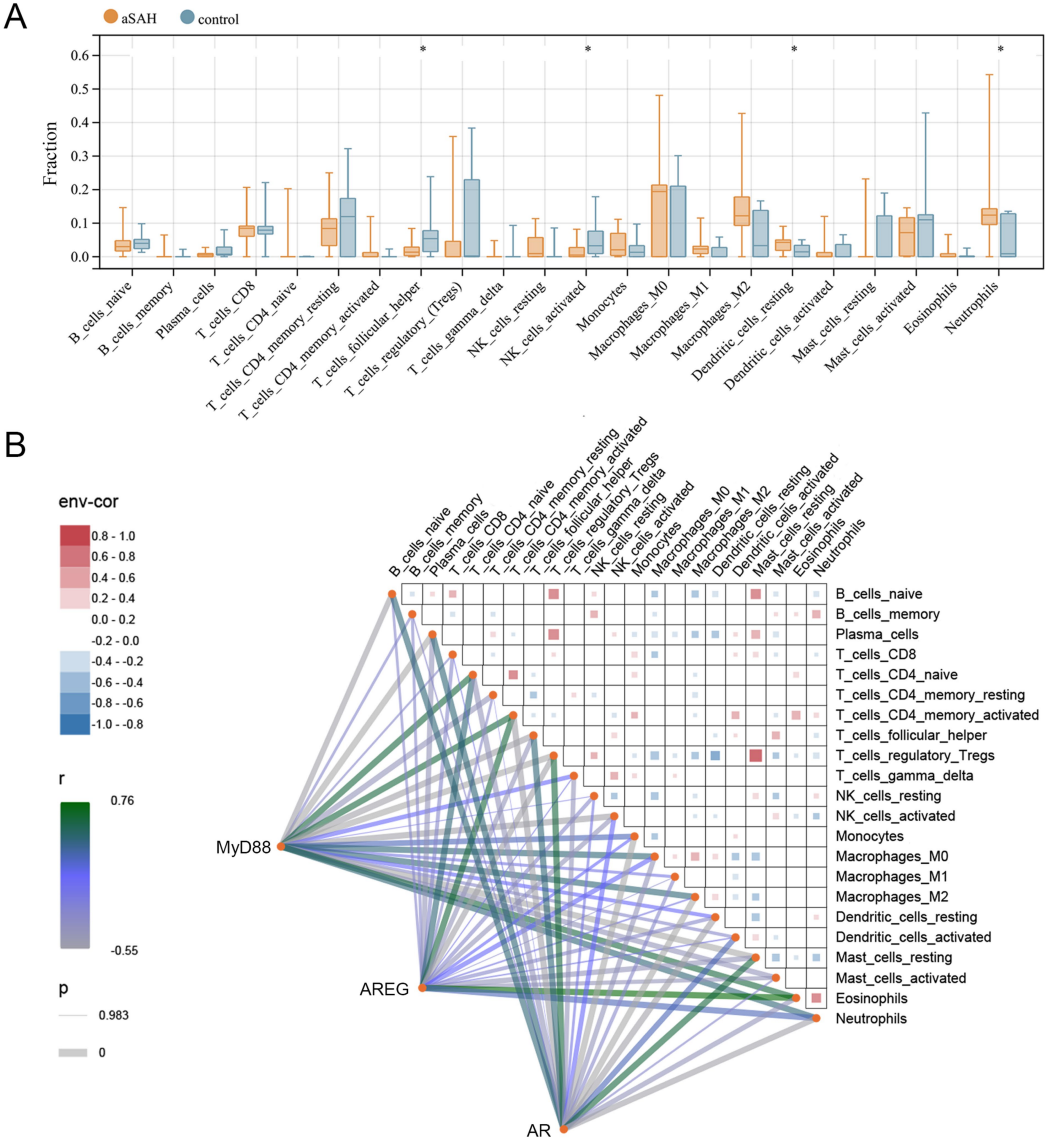
Association of hub genes with immune cells in aSAH. **(A)** Immune cell fraction between aSAH and control groups; *p < 0.05. **(B)** Correlation among immune cells and hub genes with immune cells. aSAH, aneurysmal subarachnoid hemorrhage.

Given that the KEGG analyses of both DEGs and dexmedetomidine-related targets were enriched in inflammation-related pathways, and considering the intrinsic relationship between immune cells and inflammation, we performed immune infiltration analyses to assess the differences in the infiltration levels of 22 immune cell types between the aSAH and control groups. The results demonstrated that the levels of T follicular helper cells and activated NK cells were significantly lower in the aSAH group compared to the control group, while the levels of resting dendritic cells and neutrophils were significantly higher (*p* < 0.05, Figure 4A). We further investigated the correlations between the three hub genes and these 22 immune cell types. As shown in Figure 4B, AR was positively correlated with T follicular helper cells and activated NK cells, whereas it was negatively correlated with resting dendritic cells and neutrophils. Conversely, MyD88 and AREG showed opposite correlation patterns with these immune cells, suggesting potentially distinct roles in immune regulation.

### 3.6 Hub gene-related signaling pathways

**Figure 5:**
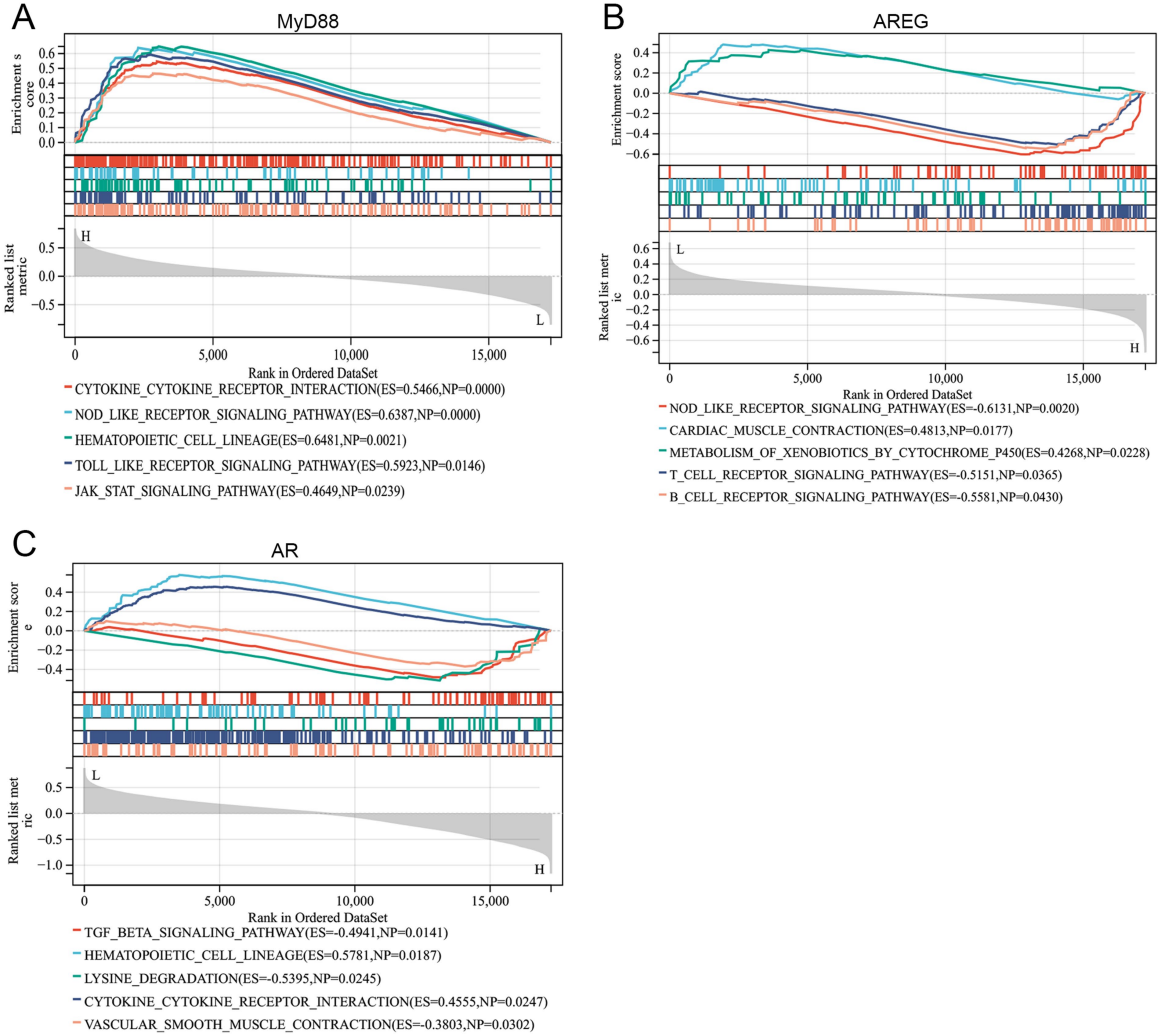
Hub gene-related signaling pathways. **(A–C)** GSEA revealed the signaling pathways related to MyD88, AREG, and AR.

To explore the underlying pathways, GSEA was conducted. The results revealed that MyD88 was positively associated with pathways such as the cytokine-cytokine receptor interaction, NOD-like receptor signaling pathway, hematopoietic cell lineage, Toll-like receptor signaling pathway, and JAK–STAT signaling pathway, indicating its potential involvement in immune signaling and inflammatory processes (Figure 5A). AREG was positively associated with cardiac muscle contraction and metabolism of xenobiotics by cytochrome, while negatively associated with the NOD-like receptor signaling pathway, T-cell receptor signaling pathway, and B-cell receptor signaling pathway, suggesting a role in metabolic and immune modulation (Figure 5B). AR was positively associated with the hematopoietic cell lineage and cytokine-cytokine receptor interaction, but negatively correlated with the TGF-β signaling pathway, lysine degradation, and vascular smooth muscle contraction, which points to its involvement in hematopoiesis and vascular regulation (Figure 5C).

### 3.7 AR had target sites with dexmedetomidine

**Figure 6:**
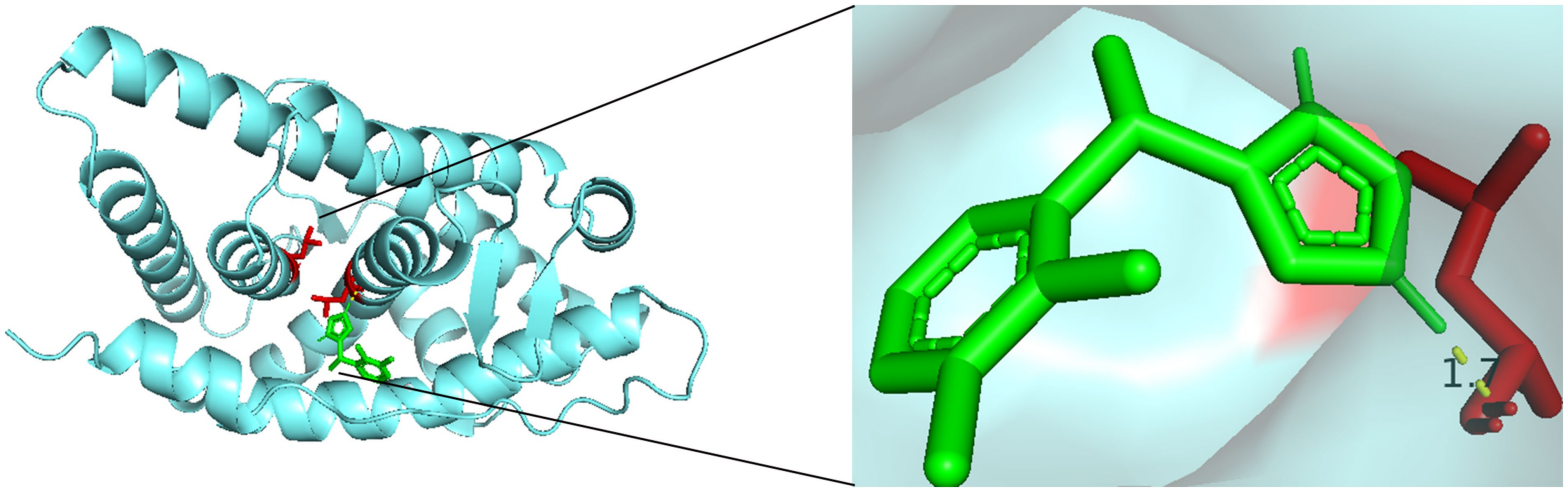
Molecular docking sites of dexmedetomidine with AR.

Finally, molecular docking analysis was performed to investigate the potential interactions between dexmedetomidine and the three hub genes. Among the three, only AR was successfully docked with dexmedetomidine, with a binding energy of −5.68 kcal/mol, suggesting a strong interaction. The details of the docking site are illustrated in Figure 6.

## 4 Discussion

aSAH is a neurological emergency that requires immediate stabilization of the patient’s condition, timely diagnosis, and treatment (16). This study comprehensively revealed the baseline characteristics, treatment-related variables, and molecular mechanisms associated with in-hospital mortality in aSAH patients. Analysis of baseline characteristics showed significant differences between the in-hospital mortality group and the non in-hospital mortality group in age, SAPS II scores, and several clinical indicators. The in-hospital mortality group had older age and higher SAPS II scores, consistent with previous findings (17). Elderly patients are more vulnerable to trauma due to frailty and chronic comorbidities, and their diminished recovery capacity imposes additional burdens (18). Furthermore, the results emphasized the importance of dexmedetomidine in reducing in-hospital mortality among aSAH patients and identified three hub genes—MyD88, AR, and AREG—as potential mediators of its protective effects. These genes are closely associated with inflammatory pathways.

In this study, both RF and XGBoost algorithms identified dexmedetomidine as the most significant treatment-related variable, with its predictive performance for in-hospital mortality (AUC) surpassing that of other treatment factors. Clinically, dexmedetomidine is widely used across various types of anesthesia procedures due to its superior sedative and analgesic properties compared to other anesthetics (19, 20). Previous studies have shown that dexmedetomidine is superior to propofol when used as the sole sedative agent during cerebral angiography in aSAH patients (21). Increasing evidence suggests that dexmedetomidine provides multiple clinical benefits in aSAH patients. For instance, it can reduce pentose phosphate pathway activity and nucleotide synthesis without impairing static cerebral autoregulation, thereby exerting neuroprotective effects in aSAH (13). A retrospective observational study found that DEX use within the first 24 h of admission was closely associated with neurological outcomes in aSAH patients (22). Additionally, it has been shown to decrease the incidence of cortical spreading depolarization and delayed cerebral ischemia in aSAH patients (23). Similarly, our logistic regression analysis confirmed the significant protective role of dexmedetomidine in reducing in-hospital mortality of aSAH patients. This finding is consistent with a recently published study that found dexmedetomidine was associated with lower in-hospital mortality in surgically treated SAH patients (24). Our results further underscore the clinical value of dexmedetomidine in the management of aSAH.

Importantly, this study identified MyD88, AR, and AREG as hub genes associated with both aSAH and dexmedetomidine. MyD88, a downstream protein in the Toll-like receptor signaling pathway, is a key mediator of inflammation. It interacts with IRAK family kinases through homotypic protein–protein interactions, ultimately activating NF-κB and MAPK pathways (25). Previous studies have shown that modulation of the MyD88-dependent pathway can effectively reduce SAH-associated neuroinflammation (25). Additionally, Cheng et al. identified MyD88 as a potential immune-related biomarker for aSAH based on analysis of the GEO database (26). *In vitro* studies have shown that inhibiting MyD88 reduces early neuronal injury after aSAH (27), and suppressing the TLR/MyD88/NF-κB axis alleviates SAH-induced inflammation (28, 29). The inhibitory effect of dexmedetomidine on this pathway has also been reported in various disease models, including acute kidney injury (30), septic liver injury (31), and neuropathic pain (32), suggesting its potential anti-inflammatory role in aSAH through the same mechanism. AR, also known as NR3C4, is a nuclear receptor involved in various pathological processes, including Kennedy disease, Klinefelter syndrome, prostate cancer, and ovarian cancer (33). Although its role in aSAH remains unexplored, AR mutations are linked to the regulation of glucocorticoid function (34). Furthermore, elevated glucocorticoid levels have been positively correlated with an increased risk of aSAH (35), while dexmedetomidine has been shown to prevent glucocorticoid-induced apoptosis of neural progenitor cells in neonatal mice (36), suggesting it may exert neuroprotective effects via modulation of the AR–glucocorticoid axis. AREG is a ligand of the epidermal growth factor receptor (EGFR). EGFR is implicated in neuronal apoptosis following SAH in mice (37), with similar findings in rats (38). Recent findings also indicate that inhibition of EGFR signaling can reduce endoplasmic reticulum stress and thereby help prevent aSAH (39). Although direct evidence of dexmedetomidine’s effect on AR and AREG remains limited, our results suggest that dexmedetomidine may exert anti-inflammatory and neuroprotective effects in aSAH by regulating MyD88, AR, and AREG. GSEA analysis supported this hypothesis, revealing associations between the hub genes and pathways such as NOD-like receptor signaling, JAK–STAT signaling, T-cell receptor signaling, and TGF-β signaling.

Inflammation is a critical pathogenic mechanism in SAH, as evidenced by neuronal injury triggered by cellular and molecular inflammation in the subarachnoid space (40). Gris et al. reported early brain infiltration and peripheral activation of innate immune cells following aSAH, highlighting the potential of immune cell targeting for treatment (41). Immunosuppression is prominent in the early post-injury phase, with reduced NK cells and regulatory T cells linked to poor outcomes in aSAH patients (42). Immune infiltration analysis in this study showed decreased levels of T follicular helper cells and activated NK cells, alongside increased neutrophils, consistent with previous reports of immune dysregulation in aSAH. Reducing peripheral neutrophils has been shown to improve neuronal injury and function (43). In our clinical data analysis, lymphocyte levels were lower, while neutrophil levels were higher in the in-hospital mortality group, underscoring the impact of innate immune cells on aSAH prognosis. Furthermore, we investigated the correlations between hub genes and immune cells. The positive correlations of MyD88 and AREG with neutrophils align with their roles in inflammatory pathways, while AR’s positive correlation with anti-inflammatory cells (T follicular helper cells and activated NK cells) highlights its potential as a therapeutic target. Previous studies have shown that dexmedetomidine may reduce neutrophil infiltration, microglial activation, and pro-inflammatory cytokine release in SAH through inhibition of the TLR4/NF-κB pathway (12). Yang et al. demonstrated that dexmedetomidine effectively maintains immune homeostasis in patients undergoing radical mastectomy, significantly increasing NK cell levels at 6 and 24 h postoperatively (44). Several studies also confirm the anti-inflammatory effects of dexmedetomidine in the nervous system (45–47). We hypothesize that dexmedetomidine exerts neuroprotective effects in aSAH by orchestrating immune cell distribution and function through multiple signaling pathways. First, by inhibiting the TLR4/MyD88/NF-κB or AREG/EGFR axis, dexmedetomidine may suppress the recruitment and activation of pro-inflammatory cells such as neutrophils, thereby alleviating local inflammation and secondary brain injury. Second, dexmedetomidine may regulate immune homeostasis through the AR-mediated glucocorticoid signaling pathway, thereby counteracting early immunosuppression and restoring the functions of T cells and NK cells.

Notably, our molecular docking analysis revealed that dexmedetomidine may directly bind to AR, suggesting a previously underappreciated potential mechanism of action. Although AR has not yet been established as a therapeutic target in aSAH, increasing evidence suggests its involvement in glucocorticoid signaling, which is closely linked to SAH pathophysiology (48–50). Currently, no AR-targeted therapies are used in clinical management of aSAH. However, selective androgen receptor modulators (SARMs) and AR antagonists have been developed and applied in fields such as prostate cancer and metabolic disorders (51–53). These existing compounds offer inspiration for repurposing or developing AR-targeted treatments in neurocritical care. We propose that the interaction between dexmedetomidine and AR may enhance its anti-inflammatory and neuroprotective effects, providing new avenues for mechanistic studies and future clinical trials in aSAH. Thus, our study not only identifies AR as a potential immunoregulatory factor in aSAH but also offers a theoretical foundation for its consideration as a novel therapeutic target.

The strength of this study lies in the integration of clinical data, machine learning algorithms, and bioinformatics data. However, we must also acknowledge its limitations. First, the study cohort was derived from the MIMIC-IV database, which may restrict the generalizability of the findings. Second, the molecular findings are based on bioinformatics analyses and require further experimental validation.

## 5 Conclusion

In summary, our research not only demonstrated a strong association between dexmedetomidine and reduced in-hospital mortality in aSAH patients but also provided mechanistic insights into the observed clinical benefits. The study identified MyD88, AR, and AREG as potential molecular targets mediating the effects of dexmedetomidine, with AR directly interacting with dexmedetomidine. These findings lay the groundwork for future exploration of the therapeutic potential and underlying mechanisms of dexmedetomidine in aSAH.

## Data Availability

The raw data supporting the conclusions of this article will be made available by the authors, without undue reservation.
